# Egocentric Representation Acquired from Offline Map Learning

**DOI:** 10.1371/journal.pone.0060194

**Published:** 2013-03-28

**Authors:** Chengli Xiao, Lei Zhang

**Affiliations:** 1 Department of Psychology, Nanjing University, Nanjing, China; 2 Department of Psychology, University of Alberta, Edmonton, Canada; McMaster University, Canada

## Abstract

It is widely accepted that people establish allocentric spatial representation after learning a map. However, it is unknown whether people can directly acquire egocentric representation after map learning. In two experiments, the participants learned a distal environment through a map and then performed the egocentric pointing tasks in that environment under three conditions: with the heading aligned with the learning perspective (baseline), after 240° rotation from the baseline (updating), and after disorientation (disorientation). Disorientation disrupted the internal consistency of pointing among objects when the participants learned the sequentially displayed map, on which only one object name was displayed at a time while the location of “self” remained on the screen all the time. However, disorientation did not affect the internal consistency of pointing among objects when the participants learned the simultaneously displayed map. These results suggest that the egocentric representation can be acquired from a sequentially presented map.

## Introduction

In modern life, we frequently consult the map before we visit a novel place. Imagine that you are driving to a shopping mall in an unfamiliar city and want to find a parking space somewhere around the mall. You would google the online map before leaving the hotel and then drive to the appropriate parking lot. After parking your car, you see a distal landmark. According to your spatial memory acquired from the map, you know that the shopping mall is on the left of this landmark. In this example, the egocentric spatial relations (e.g., knowing the location of the shopping mall with respect to the self-location when standing in the parking lot) are derived from the map. However, in most cases, your estimation of the egocentric direction of the shopping mall is not very accurate [1], [2], which may lead you to make a few mistakes (e.g., choose a wrong street or turn at a wrong corner) before finally arriving at your destination.

In a pioneer study, Thorndyke and Hayes-Roth [1] asked the participants to learn an environment either from navigation or from a map and then compared the spatial judgment performances between the two groups. The participants who studied the map were superior to the navigation group in judging relative locations and straight-line distances among objects (allocentric tasks). However, although they were able to orient themselves with respect to the unseen objects and estimate the route distance (egocentric tasks), their performance in this regard was inferior to that of the navigation group (see also [2]). It is the consensus that people maintain allocentric spatial relations after map learning [3]–[7]. When conducting the egocentric tasks, participants either transform their allocentric spatial knowledge into an egocentric one or retrieve allocentric spatial knowledge using representational egocentric frames of reference [1], [5], [8]. However, an alternative possible explanation for the above results could be that participants establish a less accurate egocentric representation after map learning than after direct experience with the environment. It is important to verify the above explanations, because they predict differently about how to improve people's egocentric performance after learning a map. If people's egocentric knowledge must be computed from an allocentric representation, the key to improve their performance on egocentric tasks lies in promoting the computing process. If people could acquire inferior egocentric representation from a map, the key to improve their performance on egocentric tasks lies in helping them establish a better egocentric representation. The goal of the present study is to examine whether people can establish an egocentric representation from a map.

The major problem of previous studies is that their egocentric tasks cannot demonstrate the presence of an egocentric representation because people can perform these tasks by either using an egocentric representation or transforming allocentric spatial relations to egocentric ones. However, the configuration error paradigm, which was first introduced by Wang and Spelke [9], can verify the presence of egocentric spatial relations. In a typical configuration error paradigm, the participants first learn the object locations in a room and then are blindfolded and are instructed to point to objects either while remaining oriented or after disorientation. The standard deviation across target objects of the mean signed pointing errors, which is referred to as the configuration error, is measured before and after disorientation, which indicates the pointing consistency among target objects in different conditions. In the egocentric representation in which objects' locations are represented with respect to the observer, the observer has to compute each individual target vector relative to him or herself during locomotion. The relative locations among different targets can only be preserved when all target locations are updated coherently over time. If this updating process is disrupted (e.g., by procedures that induce a state of disorientation), then the internal pointing consistency among all objects will decrease. Therefore, a significant increase in the configuration error after disorientation (the disorientation effect) means that the participants locate objects using an egocentric representation. On the other hand, in the allocentric representation, objects' locations are represented with respect to the salient features or the reference frames of the environment. Once preserved, these allocentric representations are no longer altered by the observer's movement, and the pointing consistency among objects, before and after disorientation, is supposed to be equivalent. Therefore, the equivalent configuration errors before and after disorientation indicate that the participants use allocentric spatial relations to locate the objects. With the configuration error paradigm, a number of studies found that participants established the egocentric and/or allocentric spatial relations after learning an environment [10]–[14].

In the present study, the configuration error paradigm was used to verify whether participants could establish the egocentric relations through a map. A you-are-here map was used to indicate each participant's location during testing and nine objects' locations around it. To make sure the participants' knowledge of the testing environment only came from the map, we adopted an offline map learning manner. After learning the map, the blindfolded participants were instructed to rotate for several circles and then guided along a 40-meter meandering way to the testing room. Since they would have lost track of the learning environment by the time they arrived at the testing room [15], the participants had already been disoriented in this case. In the testing room, the participants were tested in the configuration error paradigm, blindfolded until the end of the testing phase. When people rotated in the testing room in order to get disoriented, this was actually the second time of disorientation. To dissociate from previous experiments, we referred to the increased configuration error after disorientation as the secondary disorientation effect, which indicated the use of an egocentric representation. The absence of the secondary disorientation effect in Experiment 1 indicated that the participants acquired the allocentric spatial relations when the objects' locations were simultaneously presented on the map. However, the presence of the secondary disorientation effect in Experiment 2 provided evidence that the participants established the egocentric spatial relations when the objects' locations were displayed in a sequential manner on the map.

## Experiment 1a

The map learning phase in Experiment 1 was similar to that in previous studies [1], [2]. The participants learned the object locations in a cylindrical environment from a map. However, in contrast to the previous studies, each participant's position during testing was also shown on the map in the present research to highlight the egocentric spatial relations. After learning the map, the participants were blindfolded and escorted along a meandering trajectory to the corresponding environment, which was 40 meters away from the learning room. While standing at the testing position, the participants performed the egocentric pointing tasks with the heading aligned with the learning perspective (baseline), after 240° rotation from the baseline (updating), and after disorientation (disorientation). They were blindfolded throughout the testing phase.

### Method

#### Ethics Statement

The procedure in this study was approved by the Institutional Review Board of Nanjing University. Participants signed a written consent form before the participating in the experiment.

#### Participants

Twenty-four university students (12 men and 12 women) participated in this experiment for monetary compensation.

#### Materials and Design

**Figure 1:**
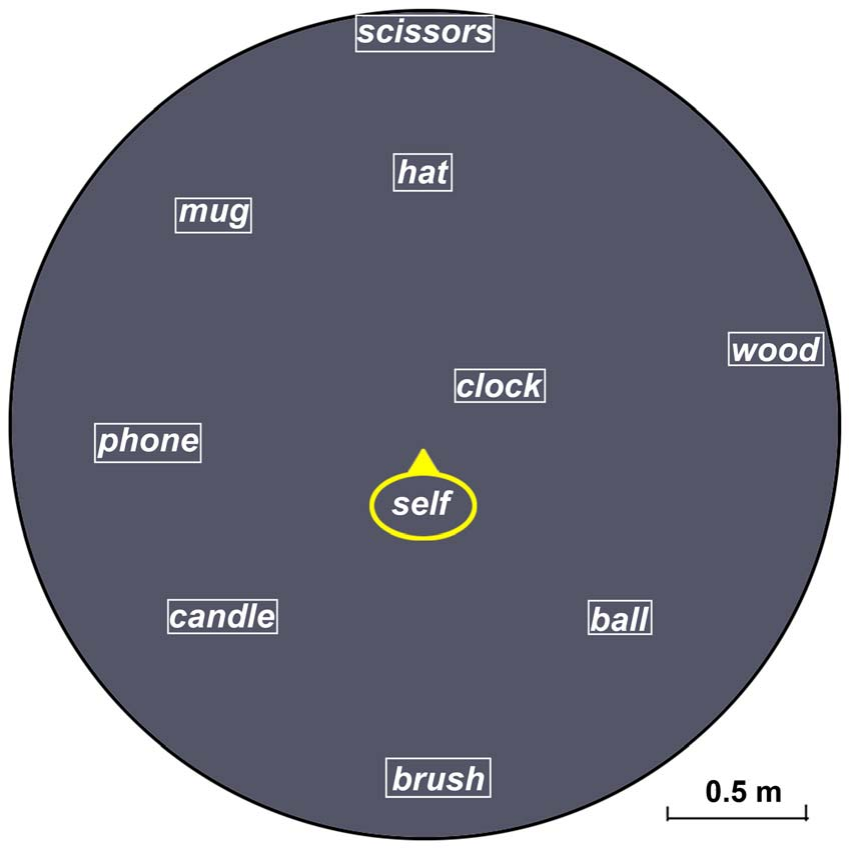
The map of the object array used in Experiment 1.

All of the learning stimuli (see Figure 1) were presented in a gray round space (diameter: 16.50 cm) on the computer, approximately 50 cm in front of the participants. The learning stimuli consisted of nine objects and one “self,” constructed in an irregular layout. The “self,” circled and marked in yellow, referred to each participant's standing position in the test. The names of the nine objects were marked in white. On the map, “scissors”, “hat”, “self”, and “brush” were in a line and parallel to the learning direction. The angles scissors-self-candle and scissors-self-ball were 120 degrees. The map was presented for 30 seconds and then masked by a black-and-white chessboard.

The testing room was 40 meters west of the learning room on the same floor and consisted of a cylindrical space 3 m in diameter circled by ceiling-to-floor curtains.

In the testing phase, the participants received instructions, including a warning indication (“start”) and target-pointing instructions (e.g., “Please point to the scissors”), through a wireless earphone connected to a computer. Then, they fulfilled the pointing tasks by using a joystick. The tasks consisted of four blocks of trials, and each block included nine trials that involve pointing to nine different objects randomly.

The primary independent variables were the three rotation conditions before the pointing tasks: *baseline*, in which the participants' facing direction was parallel to the relative direction in the learning; *updating*, in which the participants turned 240° and faced a new direction; and *disorientation*, in which the participants kept on rotating until they were disoriented. The heading directions in the updating and disorientation conditions were counterbalanced across the participants. Half of the participants faced the ball in the updating condition and faced the candle in the disorientation condition. This order was reversed for the other half of the participants. The participants were randomly assigned to each combination, with the constraint that each group contained an equal number of men and women. In each rotation condition, the participants were required to complete egocentric pointing tasks. The dependent variables were measured as in Xiao et al. [14] (see Table 1 for the definitional formulas; see also [11]). *Signed pointing error* was defined as the signed angular difference between the judged direction of the target object and the actual direction of the target object. *Heading error* was defined as the mean of the means per target object of the signed pointing error, which measured the difference between the participant's actual heading and the heading assumed by the participant while pointing. The heading error should be small because the participants were instructed that they were facing a particular object in each locomotion condition. *Configuration error* was defined as the standard deviation of the means per target object of the signed pointing errors, which would be zero if all targets moved by the same amount or would be higher if they were out of phase. We considered the configuration error as the principal measure of interest because it was the most sensitive to the pointing consistency among objects [9]; thus, we compared it between the updating and disorientation conditions. An increased configuration error after disorientation provided evidence of a secondary disorientation effect, indicating the use of egocentric representation, whereas an equivalent configuration error between these two conditions indicated the use of allocentric representation. *Pointing variability* was defined as the square root of the mean of the variances per target object of the signed pointing errors, which measured the precision of pointing judgments. However, the increased pointing variability itself could cause the increase in configuration error [9], [11], [13]. Therefore, we calculated 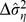 with the formula introduced by Mou et al. ([11], pp. 1286–1289) whenever both of the configuration error and the pointing variability significantly increased after disorientation. To put it simply, the analysis of 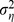 was identical to the one used to estimate power in ANOVA (e.g., [16], pp. 324–360). The configuration error (*ce*) was parallel to the mean square treatment (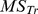), and the pointing variability (*pv*) was parallel to the mean square error (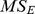). Specifically,
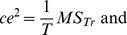


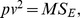
where T was the number of pointing trials per object.

**Table 1:** Definitional Formulas for Dependent Variables.

Variable	Formula
Signed pointing error for object i on trial j	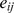 = judged direction – actual direction
Mean signed pointing error for object *i*	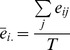
Heading error	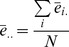
Configuration error	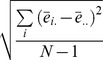
Pointing variability	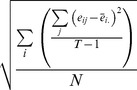

*Note:* T = number of pointing trials per object; N = number of target objects.

The contribution of inaccuracies in remembered directions of target objects (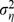) to the configuration error was
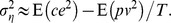



The difference between the estimates of 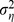 in the disorientation and updating conditions was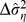. The null hypothesis was 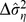 = 0, indicating that the increased configuration error was attributed to the increase in the pointing variability. On the other hand, if 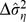 was significantly larger than 0, it indicated that the increased configuration error after disorientation was more than the artifact of the increased pointing variability. Since the pointing data were inherently directional (circular), circular statistics (see, e.g., [17]) were used to compute the dependent variables.

#### Procedure

The participants were first familiarized with the pointing task and how to use the joystick. The experimenter then showed them the real objects one by one in random order and informed them of the object names that would show on the map. Then, the participants were seated in front of a computer and instructed to study a map depicting the layout of a circular space in another room.

Followed by a fixation point, the participants viewed the map for 30 seconds. Then, they were blindfolded and asked to name and point to each object on the map with their forefinger. They had to name and point to “self” before naming and pointing to each object. This learning-pointing session was repeated 10 times.

After learning, the participants were blindfolded and led to walk outside the room. They were self-rotated in two circles in front of an open area and then guided along a meandering trajectory, walking from the learning room to the testing room. After escorting the participants to a standing position facing the scissors, the experimenter pulled up the circular curtain. In the baseline condition, the participants were informed that they were standing in the cylindrical room and at the location of “self” on the map and facing the “scissors,” parallel to the learning perspective (e.g., “Now you are standing in the cylindrical space circled by the curtain, at the location of “self” on the map and facing toward the scissors”). They were asked to put on the wireless earphone and hold the joystick at their waist and then to complete four blocks of the egocentric pointing tasks. Half of the participants were instructed to turn left until they were facing toward the ball, and the other half were instructed to turn right until they were facing toward the candle (e.g., “Please turn left until you are facing the ball”). While facing the ball/candle, the participants completed four blocks of egocentric pointing tasks. Finally, in the disorientation condition, the participants were required to rotate in place for 1 minute and then point to the location of the ball (or candle) if they faced the candle (or ball) in the updating condition. This rotating and pointing process was repeated until their absolute pointing error was more than 90°. Then, the participants were asked to turn to face what they believed was the direction of the ball (or candle) (e.g., “Please turn left until you *believe* you are facing the candle”) and complete the pointing tasks. Throughout the whole test, response accuracy was encouraged, whereas speed was not.

### Results and Discussion

The dependent variables were analyzed in repeated measures of variance analyses with one term of rotation conditions (baseline, updating, and disorientation).

**Figure 2:**
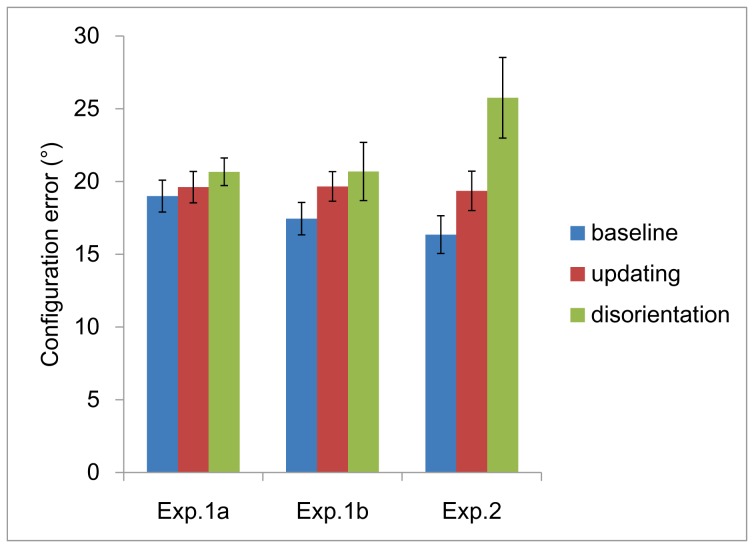
Configuration errors in Experiment 1a, Experiment 1b, Experiment 2, as a function of rotation condition and instruction. Error bars are confidence intervals corresponding to ±1 SEM.

As shown in Figure 2, there was no secondary disorientation effect after offline map learning. The configuration errors in the three rotation conditions were not significantly different, *F* (2, 46) = .95, *p* = .39, 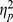 = .04.

The performances in pointing variability in the three rotation conditions were significantly different, *F* (2, 46) = 8.11, *p* = .001, 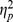 = .26. However, the procedure of disorientation did not affect the performances. Pair wise comparison showed that the pointing variability was significantly smaller in the baseline condition than in the updating and disorientation conditions, *Fs* (1, 23)≥8.40, *ps<*.008, 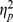
≥.27; the pointing variability did not differ in the last two conditions, *F* (1, 23) <1.

The mean absolute heading errors in the baseline, updating, and disorientation conditions were 7.34°, 17.77°, and 21.84° respectively. The main effect of the rotation condition was significant, *F* (2, 46) = 15.35, *p*<.001, 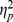 = .40. Pair wise comparison showed that the heading error was significantly smaller in the baseline condition than in the updating and disorientation conditions, *Fs* (1, 23)≥20.39, *ps<*.001, 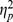
≥.47; the heading error did not differ in the last two conditions, *F*(1,23) = 2.01, *p* = .17, 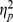 = .08. In this experiment and the following experiments, the heading errors were small in each condition, suggesting that the participants followed the instructions and believed they were facing toward the corresponding object.

In Experiment 1a, the absence of the secondary disorientation effect indicated that the participants used allocentric representation. However, Xiao et al. [14] found that people can establish both allocentric and egocentric spatial relations but primarily use one according to the regularity of the layout geometry. In addition, people can switch to update the other type of spatial relations according to the instructions. In experiment 1a, the participants may represent allocentric and egocentric relations simultaneously but may prefer to update allocentric relations primarily. Therefore, in Experiment 1b, the participants were given the same egocentric instructions as in Xiao et al. before the updating condition. If they had established the egocentric spatial relations after map learning, they should follow the instructions and use the established relations. The secondary disorientation effect should be present.

## Experiment 1b

### Method

#### Participants

Twenty-four university students (12 men and 12 women) participated in this experiment for monetary compensation.

#### Materials, Design, and Procedure

The experiment was conducted in the same way as Experiment 1a, but with one exception. Before turning toward the candle/ball in the updating condition, the participants were explicitly instructed to use egocentric spatial relations during rotation (e.g., “Please keep track of all the object locations relative to you while you are turning to face the candle”).

### Results and Discussion

As shown in Figure 2, there was no secondary disorientation effect after the offline map learning with the egocentric instructions. The configuration errors in the three rotation conditions were not significantly different, *F* (2, 46) = 1.79, *p* = .18, 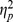 = .07.

The performances in pointing variability in the three rotation conditions were significantly different, *F* (2, 46) = 8.61, *p* = .001, 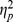 = .27. Pair wise comparison showed that the pointing variability was not significantly different between the updating and disorientation conditions, *F* (1, 23)<1; however, the pointing variability was significantly smaller in the baseline condition than in the updating and disorientation conditions, *Fs* (1, 23)≥9.10, *ps≤*.006, 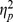
≥.28.

The mean absolute heading errors in the baseline, updating, and disorientation conditions were 4.06°, 14.36°, and 20.80° respectively. The main effect of the rotation condition was significant, *F* (2, 46) = 10.65, *p*<.001, 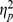 = .32. Pair wise comparison showed that the heading error was significantly smaller in the baseline condition than in the updating and disorientation conditions, *Fs* (1, 23)≥17.74, *ps<*.001, 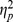
≥.44; the heading error did not differ in the last two conditions, *F*(1.23) = 2.11, *p* = .16.

Although the participants in Experiment 1b were instructed to use the egocentric relations, they did not exhibit the secondary disorientation effect, suggesting that they still used allocentric spatial relations. There was little possibility that the instruction was not strong enough because the same instruction successfully guided the participants from updating allocentric spatial relations to updating egocentric ones in Xiao et al. [14]. Moreover, Xiao et al. found that the updating of spatial relations was altered by instruction under some conditions but not for other ones. They suggested that in the later condition, the participants formed minimal, if at all, the corresponding representation in learning and merely maintained one type of representation. Thus, the participants could hardly update the representation according to the instruction. Therefore, it is very likely that the participants hardly form egocentric representation after learning a map and could only update allocentric representation despite the instruction.

Wang [18] suggests that besides the allocentric spatial relations, the egocentric snapshots, which “include all types of spatial representations that capture the state of the environment at a given moment (i.e., a “snapshot” of the environment) from the perspective of the observer,” can also lead to the absence of the disorientation effect. Since the snapshot was derived from direct interaction with the environment but not with a map, it is generally conceived that people establish the allocentric spatial relations after map learning. Thus, the absence of the second disorientation effect in Experiment 1 was regarded to be consistent with previous research that map learning leads to allocentric memory but not the egocentric snapshot.

During map learning, the participants viewed the object locations simultaneously. Although they could see the location of “self” and were required to point to the location of “self” before pointing to each object location, the allocentric relations were directly presented on the map, and the participants could pay more attention to and exert more effort toward memorizing the allocentric rather than egocentric spatial relations. However, some studies have reported that map learning is very flexible. For instance, participants form different spatial information from the same map but depending on different study goals [7]. In Experiment 2, the egocentric spatial relations were emphasized by presenting the objects' locations sequentially while showing the location of “self” constantly on the map. After map learning, the participants were blindfolded and escorted to the distal environment for testing in the configuration error paradigm.

## Experiment 2

### Method

#### Participants

Twenty-four university students (12 men and 12 women) participated in this experiment for monetary compensation.

#### Materials, Design, and Procedure

The experiment was conducted in the same way as Experiment 1a, but with one exception. In the learning phase, the participants viewed a map on which the objects' locations were presented sequentially. The “self” and the map scale were shown on the screen all the time, whereas each object's name was presented sequentially on the computer for 3333 ms in a random order. As in Experiment 1a, before turning toward the candle/ball in the updating condition, the participants did not receive explicit instruction to use the egocentric spatial relations during rotation.

### Results and Discussion

As shown in Figure 2, there was a secondary disorientation effect after sequential map learning. The participants had significantly different configuration errors in the three rotation conditions, *F* (2, 46) = 8.58, *p*<.001, 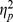 = .27. Pair wise comparison showed that the configuration error in the disorientation condition was significantly larger than that in the updating and baseline conditions, *Fs* (1, 23)≥6.76, *ps<*.02, 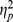
≥.23. The configuration errors in the last two conditions were not significantly different, *F* (1, 23) = 2.59, *p = *.12, 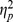 = .10.

In terms of the pointing variability, the main effect of the rotation condition was significant, *F* (2, 46) = 9.63, *p*<.001, 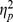 = .30. Pair wise comparison showed that the pointing variability was significantly larger in the disorientation condition than in the updating and baseline conditions, *Fs* (1, 23)≥7.09, *ps≤*.01, 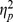
≥.24; the pointing variability did not differ in the last two conditions, *F* (1, 23) = 2.19, *p = *.15, 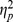 = .08. Since both of the configuration error and the pointing variability significantly increased after disorientation, and the increased configuration error might have been caused by the corresponding increase in the pointing variability, the 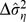 was calculate to examine this possibility [9], [11], [13]. The mean 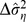 = 325.95, which was significantly larger than 0 at *t* (23) = 2.40, *p* = .03, 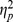 = .20, indicated that the disorientation procedure affected the internal pointing consistency among objects, not just the precision of pointing.

The mean absolute heading errors in the baseline, updating, and disorientation conditions were 5.65°, 15.38°, and 22.84°, respectively. The main effect of the rotation condition was significant, *F* (2, 46) = 13.83, *p*<.001, 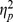 = .38. Pair wise comparison showed that the heading error was significantly smaller in the baseline condition than in the updating and disorientation conditions, *Fs* (1, 23)≥11.07, *ps*≤.003, 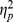
≥.33; the heading error in the updating condition was significantly smaller than that in the disorientation condition, *F* (1.23) = 5.06, *p* = .03, 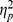 = .34.

Experiment 2 showed that the participants had a secondary disorientation effect after offline learning of the environment through a sequentially presented map, suggesting that egocentric spatial relations could be acquired through the sequentially presented map. When sequentially presented objects' locations around the location of self, the participants could only acquire self-to-object spatial relations from the map, however, when the objects were simultaneously presented around the location of self, the participants could view both self-to-object and object-to-object spatial relations. In addition, they maintained object-to-object over self-to-object ones. The object-to-object spatial relations are similar to the emergent features (e.g., [19]), which underlie the formation of object-to-object representations when all objects are presented simultaneously, but are prevented during sequential presentation. That is when certain objects can form a geometric shape such as triangle or arrow, people tends to group those objects together. In this case, when learning the map on which the objects are presented simultaneously, some objects are more likely to be grouped together and people can use the allocentric representation for reference. However, this case can never occur in sequential learning, and thus only the egocentric representation is available.

## General Discussion

The results from the two experiments with offline map learning showed that: (a) the participants established allocentric representation when the objects' and “self” locations were presented simultaneously on the map, (b) the participants established egocentric representation when the objects were presented sequentially, whereas the “self” location was constantly shown on the map.

As an exocentric learning medium, a map is believed to convey allocentric but not egocentric spatial information. On the other hand, it is believed that in most cases, the egocentric spatial relations are derived from people's direct interaction with the environment (e.g., [20]–[22]). However, the results of the present study indicate that people can also acquire egocentric spatial relations from an exocentric source and translate them into an updatable representation [23]. These findings raise two issues. First, what do people see from the sequentially presented map? If we consider self and object locations on the map as symbols, people actually view symbolic self to object locations from the map, which can be classified as symbolic egocentric. However, if we consider the “self” position on the map as a location, when learning the map, people actually view self-on-the-map to object relations. Since these relations are fixed to the environment and do not change as people moving, they can be classified as allocentric. In this study, we would refer to the self-on-the-map to object relations as symbolic egocentric relations. Second, when do people translate the symbolic egocentric codes into their egocentric coordinates? People may remember these symbolic codes in memory, and translate them into their egocentric coordinates when they test in the map corresponding environment. Alternatively, they may translate the symbolic codes into their egocentric coordinates during viewing the map, and memorize these egocentric relations. When testing, they retrieve the remembered egocentric representation. Verification of the translation time is important, because it is related to what kind of spatial memory is stored after viewing the sequentially presented map. If translation happens during map learning, it means that people store egocentric representation in long term memory. If translation happens during testing in the map corresponding environment, it means that people store symbolic egocentric representation. Further studies are needed to distinguish between above two possibilities.

Although this study provides evidence that people can acquire egocentric representation from the sequentially presented you-are-here map, it does not tell us whether this learning method can improve people's performance on egocentric tasks. There were not much difference between the performances in baseline and updating conditions of Experiment 1 and Experiment 2, and the configuration error after disorientation in Experiment 2 was even larger than those in Experiment 1. It is possible that although egocentric knowledge can be established from sequentially presented map, it is not better than that computed from allocentric representation. However, it is also possible that the map in present study is very simple, and people can easily compute egocentric relations from allocentric memory, therefore the advantage of sequential map learning is concealed. Studies with a complex environment, as the floor of a building [1], [2], comparing egocentric performances after navigation, simultaneous and sequential map learning, may address this question.

With the configuration error paradigm, Waller and Hodgson [13] replicated the finding that disorientation results in a decrease in pointing consistency among objects, and they generally interpreted this result as an indication of a switch from a relatively precise online transient representation to a relatively coarse offline enduring one. However, the presence of the secondary disorientation effect in Experiment 2 suggests that people can also retrieve the offline representation and update it online. Since the participants were tested in a room 40 meters away from the learning room, they could only retrieve their long-term memory to perform all the egocentric tasks (e.g., [24]). The superior performance in updating condition than disorientation condition suggests that the participants could retrieve the offline representations and translate them into online updatable ones, and then disrupted by disorientation. Although it is widely accepted that the online updating process is supported by the sensory-perceptual system, several recent studies have suggested that people can reinstated the online representation from their offline memory system [25]–[28]. When tested in the non-immediate environment, the instruction or the perceptual experiences affect whether the established representation can be retrieved and updated [25], [26], which may also be essential for people to successfully translate and update their map-acquired representation. For instance, in the present study, the participants were explicitly told that they were in the environment depicted on the map on their arrival in the circular layout. In addition, they could hear the curtain being pulled up, providing them with a sense of reality in the testing environment.

In conclusion, several findings of this study have broadened our understanding of the map-acquired knowledge and human's spatial updating process. In accordance with previous findings, people acquire the allocentric relations when the objects' and self positions are presented at the same time on the map. However, when the objects' names were sequentially displayed around the self position, people acquire egocentric representation. This is the first time to report that people can acquire egocentric spatial relations from an exocentric map and translate them into an updatable representation in the offline learning environment.
